# Supplementary material for: Challenges in Liver Transplantation for Hepatocellular Carcinoma: A Review of Current Controversies
Source: Cancers (Basel). 2024 Sep 2;16(17):3059. doi: 10.3390/cancers16173059 (PMC11394545; doi:10.3390/cancers16173059)
Supplement: Supplementary file 1 [file cancers-16-03059-s001.zip › cancers-3148498-supplementary.pdf]

Supplementary Table S1. Case reports and case series of patients treated with IO-based regimens before LT (updated on 23 June 2024).

Author	Year	Patients, n	Liver Disease	Milan Criteria	Treatment	ICI Duration	Time Interval between last ICI and LT	Liver Rejection	Tumor Recurrence	Death within 1 year
Nordness et al.	2020	1	HCV	Yes	N	2 years	8 days	Acute rejection, 6 POD	N/A	Yes
Schwacha-Eipper et al.	2020	1	Alcoholic Cirrhosis	Yes	N	34 cycles	21 weeks	No	No	No
Chen G. et al.	2021	1	HBV	Yes	To, L	10 cycles, unknown	93 days	Acute Hepatic Necrosis, 1 POD	N/A	Yes
Chen Z. et al.	2021	5	Cirrhosis of unknown etiology	No	N	1 cycle (3), 6 cycles (2)	59 - 122 days	No	2 Recurrences (metastatic disease)	Unknown
Dehghan et al.	2021	1	HCV	N/A	N	16 cycles	5 weeks	Subacute Hepatic Necrosis (10 POD), Graft loss, Re-transplant successful	Unknown	Unknown
Lizaola-Mayo et al.	2021	1	NASH	Yes	N, I	6 months	8 weeks	No	No	No
Qiao et al.	2021	7	Unknown	N/A	P or C + L	1 - 5 cycles	40 days	Acute cellular rejection (10 POD)	Unknown	Unknown
Sogbe et al.	2021	1	HBV	Yes	D	15 months	90 days	No	No	No

Tabrizian et al.	2021	9	5 HBV; 2 HCV; 1 NASH; 1 None	6 Yes<br>3 No	N	2 - 32 cycles	4 weeks	1 mild rejection	No	No
Schnickel et al.	2022	5	4 HCV; 1 HBV	Unknown	N	8 - 18 months	10 days - 83 months	1 Acute hepatic necrosis (14 POD), Graft loss, Re-transplant successful	No	No
Aby et al.	2022	1	HCV	No	N	23 cycles	16 days	Acute cellular rejection (9 POD)	Unknown	No
Abdelrahim et al.	2022	1	HCV	Yes	A+B	6 cycles 5 cycles	2 months	No	No	No
Kang et al.	2022	1	Unknown	Yes	P	3 cycles	138 days	No	No	No
Dave et al.	2022	6	4 HCV;<br>1 HBV;<br>1 NASH	Unknown	N	Unknown	105 days	2 rejections; Graft loss, Re-transplant successful	Unknown	1 Yes
Wang et al.	2023	16	14 HBV; 2 ALD	No	2 N<br>7 P<br>4 S<br>2 C<br>1 Multiple	1 - 27 Cycles	1 - 184 days	9 Acute liver rejection	5 Yes	Unknown
Rudolph et al.	2023	1	Unknown	Unknown	N	7 cycles	55 days	GVHD (35 POD)	No	No
Chouik et al.	2023	1	Alcoholic Cirrhosis	No	A+B	18 cycles	1 week	No	No	No

Ohm H et al.	2023	3	2 HCV;<br>1 HBV	1 Yes	2: A+B;<br>1: I+N	6-7 cycles	2 – 229 days	No	No	N/A
Abdelrahim et al.	2024	6	2 HBV;<br>2 HCV;<br>1 NASH;<br>1 Other	Both	2: A+B;<br>2: I+N<br>2: N	Variable	Median:<br>5 months	No	N/A	N/A
Guo et al.	2024	83	76% HBV<br>19.2%Other<br>4.8% HCV	32.5%<br>Yes	37.3% C+A<br>21.7% P<br>16.9% S<br>13.3% T<br>6.0% N<br>4.8% A+B	Median:<br>4 cycles	Median, (IQR):<br>58 (29-110) days	27.7%	24.1%	1-year OS:<br>88.9%

HCV: Hepatitis C Virus; HBV: Hepatitis B Virus; NASH: Non-alcoholic fatty liver disease; ALD: Alcoholic liver disease; POD: Post Operative day; To: Toripalimab; N: Nivolumab; A+B: Atezolizumab+Bevacizumab; I: Ipilimumab; P: Pembrolizumab; S: Sintilimab; C: Camrelizumab; POD: Post-operative Day; IQR: Interquartile range; N/A: Not Available

Supplementary Table S2. Case reports and case series of patients treated with IO-based regimens after LT (updated on 23 June 2024).

Author	Year	Patients, n	Liver Disease	Time Interval between LT and ICI	Treatment	IS Administered	Rejection	Death within 12 months
De Toni et al.	2017	1	HCV	1 year	N	Tacrolimus	No	No
Friend et al.	2017	2	N/A	3–4 years	N	Sirolimus	Yes	Yes
Varkaris et al.	2017	1	Cryptogenic cirrhosis	6 years	P	Tacrolimus	No	No
Gassman et al.	2018	1	HCV	2 years	N	Everolimus	Yes	Yes
DeLeon et al.	2018	5	Unknown	1.1–5.5 years	N	Tacrolimus/ Sirolimus/MMF	Yes (2)	Yes (2)
Gomez et al.	2018	1	HCV	2 years	N	Unknown	Yes	Yes
Rammohan et al.	2018	1	HBV	3 years	P	Tacrolimus/MMF	No	No
Al Jarroudi et al.	2020	3	NASH, HBV	2–3 years	N	Tacrolimus	No	No

Amjad et al.	2020	1	HCV	2 years	N	Tacrolimus/MMF	No	No
Pandey et al.	2020	1	HCV	7.5 years	I	Tacrolimus	No	No
Qui et al.	2020	1	Unknown	4 years	C	Sirolimus	No	No
Zhuang et al.	2020	1	HBV	2 years	N	Tacrolimus	No	No
Shi et al.	2021	4	Unknown	1 year	To	Sirolimus/ Everolimus	No	No
Ben Khaled et al.	2021	1	Unknown	4 years	A + B	Unknown	No	No
Yang et al.	2022	2	Unknown	1 year	A + B	Unknown	No	No

HCV: Hepatitis C Virus; HBV: Hepatitis B Virus; NASH: Non-alcoholic fatty liver disease; ALD: Alcoholic liver disease; POD: Post Operative day; To: Toripalimab; N: Nivolumab; A+B: Atezolizumab+Bevacizumab; I: Ipilimumab; P: Pembrolizumab; S: Sintilimab; C: Camrelizumab; POD: Post-operative Day; IQR: Interquartile range; N/A: Not Available; MMF, Mycophenolate Mofetil.
